# The meaning of seasonal changes, nature, and animals for adolescent girls’ wellbeing in northern Finland: A qualitative descriptive study

**DOI:** 10.3402/qhw.v11.30160

**Published:** 2016-02-22

**Authors:** Varpu Wiens, Helvi Kyngäs, Tarja Pölkki

**Affiliations:** Research Unit of Nursing Science and Health Management, University of Oulu, Oulu, Finland

**Keywords:** Wellbeing, adolescents, girls, Northern Finland, gender, seasonal changes, nature and animals

## Abstract

Wellbeing is complex, holistic, and subjectively perceived. Issues such as gender, age, and environment seem to affect it. Therefore, the aim of this qualitative study was to describe the meaning of seasonal changes, nature, and animals towards 13–16-year-old girls’ wellbeing in Northern Finland. In the spring of 2014, through purposive sampling, a total of 19 girls participated in semi-structured interviews from various parts of Northern Finland. The data were analysed using content analysis. Afterwards, the analysis combining the category *participatory involvement with environment* was found, and this consisted of three main categories: adaptation to seasonal changes, restorative nature, and empowering interactivity with animals. Seasonal changes had an effect on girls’ wellbeing; in the summertime, they felt happy and vivacious, active, and outgoing. Instead, during the winter months, girls’ mood and activity seemed to be lower and they felt lazier and depressed. Nature brought mainly positive feelings to girls; being in nature was experienced as liberating and relaxing, and it offered opportunities to relax and have sensory perceptions. Interaction with animals was perceived as empowering. They were experienced as altruistic and comforting companions. Animals were important to girls, and they contributed to girls’ lives through positive effects towards their mental and physical wellbeing. Based on the results of this study, we can recommend that being in nature and interacting with animals should be supported because they seem to have benefits towards adolescent girls’ health and wellbeing. In order to facilitate the negative effects of winter, the school days should be arranged in such a way that it would be possible for girls to have outdoor activities during the daytime. The challenge for the future is perhaps the purposeful utilisation of nature's and the animals’ positive effects towards their wellbeing.

Wellbeing is a holistic experience that is composed from different sources and is seen as a subjectively perceived process. Wellbeing is influenced and affected by issues that are beyond the health sector domain. The past, present, and future also play an important role in how wellbeing is perceived, not to mention the genes and environment that are closely tied, and both have an equal impact on wellbeing (Polderman et al., 2015). When researching wellbeing, it is good to look at both negative and positive sides; an important aspect of wellbeing is one's overall evaluation of life or life satisfaction (Torsheim, Aaroe, & Wold, 2001) and to involve the target group (World Health Organization [WHO], 1986). Because adolescence is a time of depression and some anxiety disorders (Costello, Copeland, & Angold, 2011), all aspects should be taken into account to support wellbeing. Among other things, gender acts as a critical determinant of health, thus influencing health and health experiences, research, and practice (Gelb, Pederson, & Greaves, 2011). Girls present different and, to some extent, more symptoms and seem to perceive their health at some point as poorer than boys (Derdikman-Eiron et al., 2011; Jerdén, Burell, Stenlund, Weinehall, & Bergström, 2011). Also, Landstedt, Hammarström, and Winefield (2015) found in the Northern Swedish Cohort study that girls or women reported higher levels of internalising symptoms and functional somatic symptoms than boys or men. In the study from Kekkonen et al. (2015), the female gender was associated with frequent primary healthcare use. The Finnish School Health Promotion study results from 2010 and 2013 (Finnish National Institute for Health and Welfare, 2013) indicated that girls living in Northern Finland had more problems related to alcohol consumption and experienced more sexual violence than girls living in other parts of the country. They also experienced their health status as mediocre or weak, felt tired almost every day, had neck and shoulder pain on a weekly basis, had headaches on a weekly basis, and experienced depression. Rönkä, Taanila, Koiranen, Sunnari, and Rautio (2013) noted in their study that more girls than boys reported deliberate self-harm in the population from the Northern Finland Birth Cohorts of 1986.

The meaning of life in the North manifests in the diverse everyday and ubiquitous activity of life, in which there is darkness or extreme light, harsh weather conditions, distances, reduced service availability, and a reduction in the people. However, everyday life in Northern Finland is good enough and beautiful despite the challenges (Rautio, 2011). Finland is one of the northernmost countries; it lies approximately between latitudes 60° and 70° N and longitudes 20° and 32° E. Finland's climate is characterised by seasonal changes, and during the winter season, it is cold and dark (Finnish Meteorological Institute, 2015). Finland's annual average temperature varies from the Finnish southwest part of the rich five degrees to Northern Lapland a couple of centigrade zero and the least sunshine is obtained in the eastern part of Lapland, about 1300 h/year (Pirinen et al., 2012). Earlier studies have shown that seasonal changes affect mood and behaviour, and girls living in the 67th latitude reported more seasonal distress than girls living in the 60th latitude. Seasonal alterations in child and adolescent behaviour are not well understood and must be investigated more thoroughly (Rastad, Ulfberg, & Sjödén, 2006; Sourander, Koskelainen, & Helenius, 1999). It was shown in other studies that girls were physically less active and spent more sedentary time during winter (Gracia-Marco et al., 2013) and that the female gender was affected more sensitively to seasonality in mood (Tonettia, Barbatob, Fabbria, Adanc, & Natalea, 2007). In the study of Kristjánsdóttir, Olsson, Sundelin, and Naessen (2013), adolescent girls showed seasonal variations in self-reported health and depressive symptoms with more symptoms during winter months and, thus, in their opinion, the high prevalence of suspected depression during the winter months deserves attention. Konu, Joronen, and Lintonen (2015) found in their study that pupils perceived their wellbeing highest during the period lasting from the middle of October until the end of December, and the health status was perceived lowest from the middle of March to the end of May. On the other hand, Rastad et al. (2006) found that depressive symptoms during autumn and winter were common among Swedish senior high school students, especially among girls.

Even if Lee and Maheswaran (2010) found there was weak evidence for the links between physical and mental health, wellbeing, and urban green space, there are studies that have shown that green areas are connected to better perceived health (Maas et al., 2009; Tamosiunas et al., 2014; Tsunetsugu et al., 2013; Tyrväinen et al., 2014) and that repeated exercise in nature is connected to better emotional wellbeing (Pasanen, Tyrväinen, & Korpela, 2014). Landscapes have the potential to promote mental, physical, and social wellbeing in many ways: through attention restoration, stress reduction, and the evocation of positive emotions; promotion of physical activity and social integration; and social engagement and participation (Abraham, Sommerhalder, & Abel, 2010). Findings from Taylor, Kuo, and Sullivan (2002) suggested that, for girls, the green space immediately outside the home can help them lead more effective, self-disciplined lives, and Wells and Evans (2003) noted that the impact of life stress was lower among children with high levels of nearby nature than among those with little nearby nature. Alongside nature, animals also appear to affect their wellbeing (Zilcha-Mano, Mikulincer, & Shaver, 2012). For instance, equine-facilitated interventions on human wellbeing may contribute to adolescents in various ways (Hauge, Kvalem, Berget, Enders-Slegers, & Braastad, 2014; Pendry, Smith, & Roeter, 2014). The results from Endenburg and Lith's (2011) findings suggested that animals positively influence children's development and have a valuable role in therapy.

In some respects, the voices of adolescents are still not heard in terms of the matters concerning them (Carral, Braddick, Jané-Llopis, & Jenkins, 2009). Listening to adolescents is recommended by The United Nations Children's Fund's (UNICEF's) Convention on the Rights of the Child, where children have the right to have their say in matters that concern them (The United Nations, 1989). As Borg, Salmelin, Joukamaa, and Tamminen (2014) stated, listening to adolescents and then utilising their vision and experience provide us the possibility to intervene more specifically and sooner. The findings from our earlier study indicated that girls seemed to appreciate the positive effects of natural environments, and it was important to deepen the understanding of this meaning. To the participating girls, wellbeing was a positive experience and feeling that was revealed when they interacted between their relationships, living conditions, lifestyle, and environment (Wiens, Kyngäs, & Pölkki, 2014).Thus, the aim of this study was to describe the meaning of seasonal changes, nature, and animals towards 13–16-year-old girls’ wellbeing in Northern Finland. In the context of this study, “meaning” is what relevancy, substance, and the significance of seasonal changes, nature, and animals have towards adolescent girls’ wellbeing based on their experiences. Obtaining information from the adolescents themselves is meant to implement the recommended notion and thus gain more of an understanding and vision about their local daily lives and needs.

## Materials and method

### Ethical considerations

The study was approved by the Northern Ostrobothnia Hospital District Ethics Committee before it began. Throughout the study, attention was paid to human dignity, which includes participants’ consent and voluntariness and maintaining confidentiality. Written consent was obtained from participating girls and their parents. Before the start of the interview, girls were again informed that they could withdraw from the study anytime if they wanted to. The participants’ confidentiality was ensured by assigning ID numbers to their interviews that could not be linked back to their identities and by meticulously quoting participants’ statements from the interview in the text. Furthermore, the submissions were available only to the researchers.

### Data collection and participants

A semi-structured interview was chosen to ensure that all required information was obtained and provided the people the freedom to provide as many illustrations and explanations as they wished (Polit & Beck, 2012). Before the start of the study, a preliminary interview was carried out to ensure the intelligibility of the interview themes, and minor changes were made regarding phrasing. Purposive sampling was used to enhance information richness (Polit & Beck, 2012). Data collection started by contacting youth counsellors, teachers, and sports or hobby instructors from various parts of Northern Finland who had contacts with 13–16-year-old girls. In their own living area, these contact persons made inquiries to the girls they knew who would like to participate in the research. Information, instructions, and consent forms were sent in advance to the contact persons to be handed to the girls and their guardians. Contact persons also concurred with the girls of the date and location where the interviews would take place. On the appropriate date, the researcher travelled to the various parts of Northern Finland, verified the signed consent forms, and completed the interviews. Although the girls had arrived to the site and filled and signed the consent forms, the researcher still informed them about the voluntary participation before the data collection. So, material was obtained from 19 girls aged 13–16 living in various parts of Northern Finland who gave their consent. Interviews were conducted between February and May in 2014. To gain as authentic and versatile descriptions as possible, the criteria used in the selection of the participants ensured that they were 13–16 years old and lived in Northern Finland. The average age of the interviewed girls was 14.4 years old. Almost all of the girls who were interviewed resided in Northern Finland, lived in rural areas or villages, or lived in small towns or an area of dispersed settlement. During the interviews, it emerged that most of the girls interacted with animals on a regular basis.

The interview locations mainly consisted of local youth centres as well as school premises, recreational places, or homes. The total time of all interviews was 406 min and 28 s. The duration of the interviews ranged from 10 min and 17 s to 35 min and 12 s. Interviews were conducted until data saturation was obtained. The interview had three themes: 1) seasonal changes, 2) nature, and 3) animals, and the meaning of these subjects towards the girls’ wellbeing was discussed.

### Data analysis

**Figure 1:**
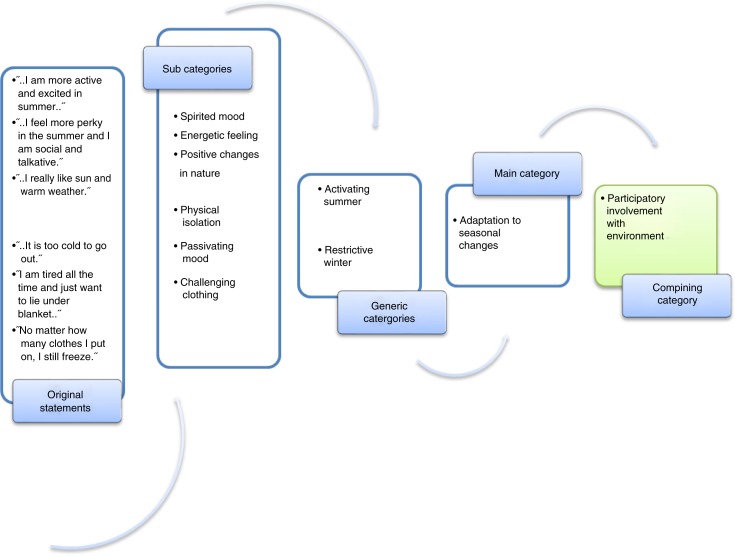
Illustration of the data analysis process showing the grouping of subcategories to form the main category, “adaptation to seasonal changes.”

Content analysis was chosen as the analysis method for the interviews because it represents a systematic and objective means of describing and quantifying phenomena (Elo & Kyngäs, 2008; Schreier, 2012). The interviews were grouped according to themes and inside the themes, the analysis was preceded inductively using the content analysis. The analysis began by listening to the recorded interviews, and after listening, the interviews were transcribed. The transcription was done by the same researcher who also did the interviews to avoid possible misspelling, omission of words, or misinterpretation. In the first stage of the analysis, each girl's interview was read from beginning to end several times to ensure that the researcher had a clear grasp of its overall contents. After becoming familiar with the data, the data organisation began with open coding. Notes and headings were written down and collected to the coding sheets. The units of analysis included words or statements that related to the same central meaning. Girls’ original expressions were then reduced to convert them into simplified expression. Expressions with similar meanings or dealing with related topics were then grouped into subcategories. Subcategories with similar meanings were then grouped to form generic and main categories, which were named using content-characteristic words (Elo et al., 2014). This abstraction process was continued until new categories could no longer be formed. A combining category was named to designate a common descriptive name for the main categories; as Cavanagh (1997) noted, the purpose of creating categories is to provide a means of describing the phenomenon, to increase understanding, and to generate knowledge. One researcher (V.W.) had responsibility for the data analysis, and after forming the categories, they were discussed and defined in the research group (Fig. 1).

## Results

**Figure 2:**
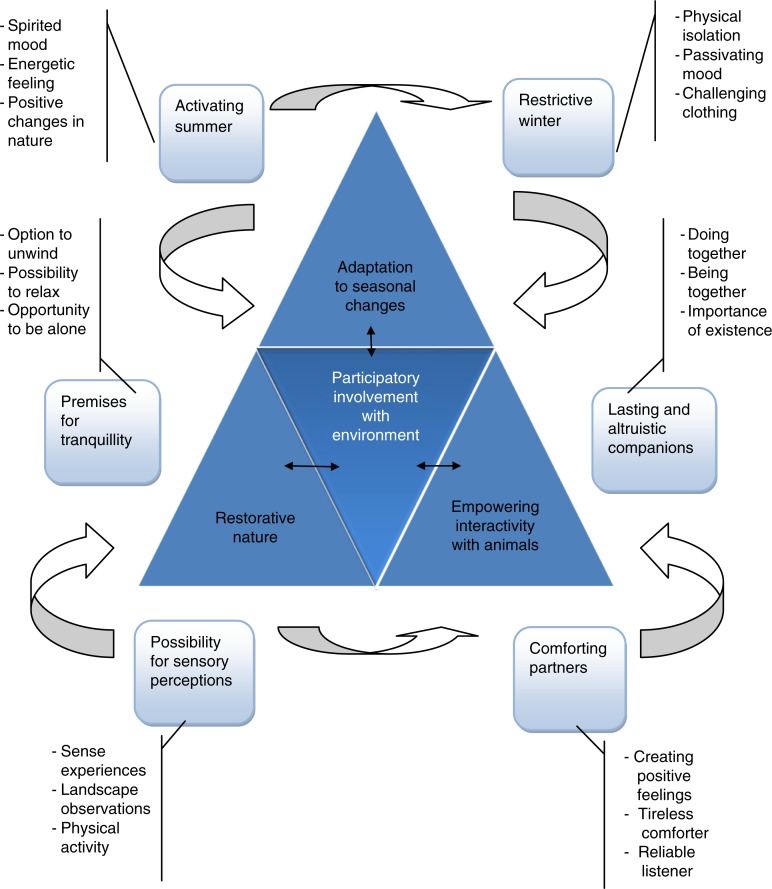
The bonding meaning of the environment.

Afterwards, the analysis combining category *participatory involvement with environment* was found, which consists of three main categories: adaptation to seasonal changes, restorative nature, and empowering interactivity with animals (Fig. 2). The elements in these categories incorporate (seasonal changes, restorative nature, and empowering animals) common meanings to the girls, and they are related to each other. The elements in these categories cause girls, willingly or unwillingly, to take part in the environment and its changes, and generate feelings and emotions, thus providing meaning to their wellbeing.

### Adaptation to seasonal changes

Two generic categories were found: activating summer and restrictive winter. Seasonal changes and their variations had an important meaning towards girls’ wellbeing. In summer, girls felt they were happier and vivacious, active, and outgoing. Whereas, during the winter months, girls’ mood and activity seemed to be lower, they moved around and went out less, and they feel lazier and more depressed.

### Activating summer

Activating summer consisted of subcategories that included spirited mood, energetic feeling, and positive changes in nature. In the summer, girls felt they were more cheerful and happy. They felt that summertime was nicer than winter because people in general were more active and cheerful than in winter. Even attending school seemed to be easy and effortless. In the summertime, girls felt more social and less lonely; they met friends in spontaneous ways and, in general, had more things to do than in winter. Girls spent much of their time indoors in the winter, whereas, in the summer, a lot of activities took place outdoors. Sun and warm weather were important to girls. In the summer, they felt like they could cope more and do more versatile and diverse activities things. And the fact cannot be forgotten that summer is mainly a holiday season when exciting things like travelling and meeting new people occur.

Well, in the summer we are more on the go all the time, because weather allows us to drive mopeds or bikes, and this enables us to go away from the small villages, and then there are shores and places outdoors where you can spend time and you do not have to find a place inside like in wintertime. (Interview 5)

In summer, girls were sprightly, felt energetic, and they moved around more and met friends. They felt that they were able to do versatile things and had the energy to carry them out. In the summer, it is lighter, and darkness does not restrict mobility. This energetic feeling seemed to proceed at the same pace with the onset of spring. They also felt that time went faster in summer than in winter. Girls felt that it was easier to do different practical things such as wearing fewer clothes, which made life more pleasant. But there was one unpleasant thing about summer, namely the mosquitoes.

Uh, I might like to move around more in summer. I have many hobbies in the winter too, but in the summer, there is the possibility to take a swim and such. I feel energetic and have a feeling that I have energy to do things. (Interview 12)

### Restrictive winter

Restrictive winter consisted of subcategories that included physical isolation, passivating mood, and challenging clothing. Winter and issues related to it were mainly mentioned in a negative tone. Coldness and darkness had a restricted meaning to impact girls, and they did not want to go outdoors because of them. Both these elements and the lack of light and warmth seemed to reduce the experience of wellbeing. The girls missed the sun during winter, and being in the dark made them feel depressed, bored, and apathetical. Being and living in the dark also made girls feel tired and reluctant. They also mentioned being snappy and irritable, and this was partly the result of being passive and spending too much time indoors.

Umm, it affects me in a way, that in the winter when we have the polar nights and it is really dark, I feel depressed and I don't want to get off the couch and I don't want to do anything. (Interview 15)

Well, I do not know, I am tired and just want to lie under the blanket and watch television; just too lazy to do anything. (Interview 8)

Finding the right clothing was difficult; they either had too many clothes, which made them feel too hot and it was difficult to move, or they had insufficient clothing and were freezing. In addition, the challenge was to find a balance between out- and indoor clothing when moving between indoor and outdoor spaces; for example, moving between home and school. Feeling cold and feeling the frost were considered uncomfortable, so when possible, girls stayed indoors. A temperature between −20 and −30°C was considered too cold and inconvenient to go out. Walking in the darkness was experienced as uncomfortable and gloomy, so this also reduced the incentive to go out. Wintertime also affected girls’ school attendance, and some of them felt indifferent about it. Even the time and days seemed to pass slower in the wintertime. Just being indoors, watching television, lying under covers, and having a lack of energy irritated girls, which made them feel even more passive, withdrawn, and uncommunicative.

Eh, when it is frost, there is this problem; when I go to school and want to walk, I am having problems with clothing, and I hate it when I am freezing, and when dressed well, I can't move properly. (Interview 3)

Well, I am inside a lot more, and then I am, like, not so social, and then there is this school, and it is, like, I could not care less. (Interview 9)

Although wintertime was mostly felt as restrictive, its positive side was snow, which enabled winter sport activities such as skiing, skating, and snowmobiling. Usually these winter activities were practised more at the end of winter and closer to spring.

There are different winters, the snowdrift is nice, you can walk or ski on it, and then when it is soft, you think and laugh at yourself, what was the point when you sink in, but it is funny … (Interview 13)

### Restorative nature

Two generic categories were found: premises for tranquillity and the possibility for sensory perceptions. Being in nature mainly provided the girls with positive feelings; it was experienced as liberating and relaxing. Nature offered the opportunities to calm down and relax as well as observe and have sensory perceptions.

### Premises for tranquillity

The premise for tranquillity consisted of subcategories including the option to unwind, possibility to relax, and opportunity to be alone. When by themselves, they had the opportunity to ponder and go through their thoughts. Moving around in the forest was perceived to be liberating, and having the chance to be alone was valued. The girls, in particular, appreciated the possibility of being alone; it seemed that the girls were looking for silence and peace. Animals, plants, and trees were important parts of the experience of silence. Most of the girls liked moving in an unkempt forest, while few enjoyed maintained parks. There were some negative issues that had disturbing elements, including manmade damage, clutter, and the sound of machines. Sometimes, moving in the forest was intimidating because of the darkness or frightening animals.

Whenever I get to go out in to the nature, if I am feeling down, all my worries will go away and I get into a good mood because I don't have a care in the world. I just think about the nature and everything that is related to it, and this gives me a positive spirit. (Interview 12)

Dwelling in nature provided the feeling that worries were disappearing, the concerned mood was replaced with good humour, and the girls felt more positive. At times when something was irritating or stressing, or the situation at home was noisy or otherwise difficult, going out into nature was experienced as being soothing. In nature, the girls could set their minds at ease; they had the choice to think and be left alone. Sometimes, a friend or a dog accompanied the girls on their walks. But even when sharing the walk in the nature with a friend, it was not mandatory to talk. To the girls, walking in the forest offered the possibility to unload energy or a bad mood.

So, I don't know, when I get irritated and so on, it is nice to go for a walk somewhere where it is peaceful and there is nobody, I just like to walk … I don't know, it is liberating, or whatever the word for it is …. (Interview 19)

### Possibility for sensory perceptions

The possibility for sensory perceptions consisted of subcategories including the sense experiences, landscape observations, and physical activity. Sensations in nature, observing the landscape, and actual physical acts like touching and smelling were parts of this perception. The entity of these experiences consisted of sounds, colours, temperatures, light, and animals; thus, it offered something for all of the senses. A feeling of familiarity was mentioned; the girls had been accustomed to the forest since childhood; thus, the environment was perceived as being mainly safe. The girls could walk, sit, or lie down in the forest. Actual physical acts included touching trees and different natural materials, as well as picking berries. Green colours, bright stars, and identifying familiar smells, sounds, and materials were felt to be rewarding and pleasant. It was important to the girls that the forest was in its natural state—this, in their mind, enabled diverse scenery (e.g., dense forest or swamps), with different plants and animals. In particular, trees were important. Sometimes, just sitting down and looking into the distance calmed and appeased them.

Because it is quiet and you can see different animals and it is beautiful and then there is, I don't know, well, I feel so liberated, because there is nobody and nothing that bothers me, and I can be in peace and undisturbed. (Interview 16)

Well, [there are] colours in nature and green trees, and it is quiet, and then you sometimes see some animals, birds, and little bugs and bees. (Interview 7)

### Empowering interactivity with animals

Two generic categories were found: lasting and altruistic companions and comforting partners. Animals were important to the girls and contributed to the girls’ lives. Animals and pets had positive meanings for mental and physical wellbeing. The animals mentioned included dogs, cats, horses, and reindeer.

### Lasting and altruistic companions

This consisted of the subcategories of doing things and being together, and the importance of existence. Animals neither judged nor criticised the girls, and being with the animals provided immediate, positive, and honest feedback. With animals, the girls could just be themselves; they did not need to pretend or present anything they were not. Animals gave companionship and were nice to have around because they stimulate and comfort at the same time. The girls became attached to the animals, and being with them helped the girls to think about what it is to be responsible for another being. And thus, taking care of animals helped the girls take care of themselves too. The girls came into contact with nature through animals, both by the animals themselves and when going out into nature with them. Doing activities together with animals in nature (e.g., riding horses or walking with dogs) was rewarding. They also gave learning experiences and insights. But animals were important for girls just by being there.

Well, they make me happy, and I have to take care of them. And when I nurse them, I feel good myself too; they trust me. And then I spent time outdoors, not just inside like others—they just waste time with phones because they don't have animals and they don't go out at all. But I have to; I just can't ignore it. And when I go out, my mind widens and I forget everything. (Interview 4)

To the girls, their relationships with animals were important—sometimes even the most important thing. The presence of animals brought comfort; the animals were refreshing and stimulating. The mere essence of the animal brought positivity to the girls’ lives, and they just liked to watch the animals bustle around. The girls did not feel alone when they had pets, and animals helped them through rough times. They were even considered substitutes for friends. This unconditional love that pets and animals gave to the girls was important. And if the girls had the feeling that the animals trusted them, it gave them a feeling of success and energy.

Oh, well, they are like, you know that they are there for you always and just for you. For example, at least your own pets love you and they are close to you. (Interview 8)

### Comforting partner

This consisted of the subcategories of creating a positive feeling, tireless comforter, and reliable listener. The mere presence of animals made the girls feel positive. Animals were perceived as being invigorating and bustling, and moving with them was experienced as stimulating. The girls’ wellbeing was enhanced because they could talk to the animals about their concerns safely and freely. Animals were considered reliable listeners and untiring comforters, and they gave the girls the feeling that they were on their side in matters.

I don't know, you really get attached to them, and I can tell my dogs everything, even things I couldn't tell anyone else, ever. Then, I get this feeling that there is at least someone I can talk to if I can't tell these things to anyone else. (Interview 10)

The girls were touched by the animals’ altruism, and in return they wanted to take good care of the animals. When the girls were feeling sad or reluctant, pets gave them consolation. The same phenomenon happened when the girls came home from school to an empty home, as there were at least pets to welcome them warmly and happily, which made the girls also feel happy. If the girls were experiencing emotional difficulties, animals helped girls to forget their sorrows for a while, just with their presence. In addition, animals also had a calming effect on the girls. One of the most important realisations that animals gave to the girls was the notion and understanding that, by taking care of animals, they learned to take care of themselves too.

It is good to know you are important to someone, and they really make me happy and in a good mood, and my two cats have truly helped me when I was experiencing tough times. (Interview 9)

## Discussion

This study aimed to describe the meanings of seasonal changes, nature, and animals for the wellbeing of girls aged 13–16 in Northern Finland, as described by them. One premise of this study was to implement the thought of obtaining information from young people themselves in matters affecting them. On the basis of the results, it seems that the environment has a bonding and participatory meaning to girls’ lives, with both enhancing and reducing effects to their wellbeing. Restorative nature, adaptation to seasonal changes, and empowering animals have common denominators that seem to have a meaning to girls’ wellbeing in a way where they must respond and adapt to circumstances and events. For example, these responses are manifested by being more active or passive, thus increasing or decreasing the intensity or contact of the issues that have a meaning to their wellbeing in Northern Finland. There are also differences between the meaning of seasonal changes, nature, and animals toward wellbeing. It seems that girls could regulate more issues dealing with nature and animals, but seasonal changes are a relatively stable phenomenon, and their effects cannot be eased or mitigated easily. The environment and its events make the girls unwilling or willing partakers, which occurs regardless if they wished for it.

Nature seems to offer girls the means to regulate their wellbeing through tranquillity and perceptions. In this study, girls described how nature affects them through different senses; these were observations through looking, touching, hearing, and smelling. Importantly, they could not hear noises because silence was perceived as an important sensation. This was one of the things that offered the opportunity for the girls to unwind and relax, as Korpela, Borodulin, Neuvonen, Paronen, and Tyrväinen (2014) state, referring to the importance of experiencing calmness, gaining new spirit and vitality for everyday routines, forgetting everyday worries, clarifying one's thoughts, and gaining faith in tomorrow during nature-based recreation. In the study of Pálsdóttir, Persson, Persson, and Grahn (2014), *social quietness* was an important component that facilitated personal and intimate engagement with the natural environments. Although their study was not related to adolescent girls, this *social quietness* is in line with the results from this study's finding, where the girls looked for options to unwind and relax and opportunities to be alone in nature. More often than not, the girls did not describe being scared or afraid in nature, but when they did, it was in contexts with darkness. This was probably linked to the fact that the girls were moving within familiar surroundings in nature. The girls enjoyed physical activity in nature alone or with friends, and perhaps these various occasions had different functions on their wellbeing. Other studies have also shown that repeated exercise in nature is connected to better emotional wellbeing (Annerstedt & Wählborg, 2011; Bratman, Hamilton, Hahn, Daily, & Gross, 2015; Mottaa, McWilliams, Schwartz, & Cavera, 2012; Pasanen et al., 2014). In this study, the opportunity to be alone was also perceived as being important, and it would be interesting to determine if this feature occurs only among Finnish girls living in Northern Finland. The girls described that nature offered possibilities for sense experiences, landscape observations, and physical activity. But most importantly, when moving in nature, they described that they had the opportunity to be themselves. The possibility of experiencing such a feeling, one would think, is relevant in these times, when girls face a lot of external pressure to be acceptable. We share the findings of our study with those of Russell et al.'s (2013) research—that knowing and experiencing nature generally make people happier and healthier people—and with those of Abraham et al. (2010)—that landscapes have potential as a resource for physical, mental, and social wellbeing. Since wellbeing is enhanced in nature, in residential areas there should be sufficient green areas close to home (Maas et al., 2009). Children should also be encouraged to go outside into nature. As Bratman et al. (2015) note in their study, accessible natural areas may be vital for mental health in our rapidly urbanising world.

Animals have a positive impact on human wellbeing, affecting psychical health, mental wellbeing, and social relations (Lass-Hennemann, Peyk, Streb, Holz, & Michael, 2014; Pendry et al., 2014; Polheber & Matchock, 2013; Virues-Ortega & Buela-Casal, 2006). This also emerged in this study as the girls felt that animals gave them comfort and companionship, and perceived animals as being consoling. Animals had calming and invigorating effects at the same time, and it seems that animals had the ability to respond to the girls’ emotional needs properly. This fits with previous research showing that interaction with animals, and even more so with one's own companion animal, can alleviate endocrinological and cardiovascular stress responses (Beetz, Julius, Turner, & Kotrschal, 2012; Cole, Gawlinski, Steers, & Kotlerman, 2007; Motooka, Koike, Yokoyama, & Kennedy, 2006; Viau et al., 2010). According to Odendaal and Meinjes (2003), interaction with a familiar dog in the home has been found to decrease human cortisol levels after as little as 3 min of physical interaction with a dog. Zilcha-Mano et al. (2012) found in their study that physical or cognitive presence of a pet provided to owners’ feelings of competence and a secure base from which they could entertain ambitions and experience a greater sense of self-efficacy.

In addition to smaller pets like dogs and cats, horses also seem to have positive meaning to girls’ wellbeing (Burgon, 2011; Forsberg & Tebelius, 2011; Trotter, Chandler, Goodwin-Bond & Casey, 2008). In this study, among the girls who interacted with horses, horses had central importance to them and thus also for their wellbeing. As noted by Granados and Agís (2011), hippotherapy affects multiple systems in humans simultaneously, such as the sensory, muscular, skeletal, limbic, vestibular, and ocular systems, which leads to psychological, social, and educational benefits. The indication that the interaction between horses and young people may have positive physiological, physical, and psychological effects opens up the possibility of using horse-assisted activities to promote health and prevent illness. The practical implications of the study from Hauge et al. (2014) show that offering stable work and riding to adolescents in an environment with a supportive adult and peers may benefit their psychological development.

One focal observation from this study was that the girls described how animals did not judge or criticise them, and they were tireless comforters and reliable listeners. Being with animals gave them immediate, positive, and honest feedback. As Wood et al. (2015) stated, supporting pet ownership may well be an under-recognised conduit for individual and community wellbeing. According to Sable (2013), there is scientific evidence that companion animals have positive effects on humans’ psychological and physical wellbeing. It appears that companion pets are able to act as buffers against the stresses of daily life, offering a degree of unconditional social support (Eachus, 2001).

According to this study, seasonal changes and variations seem to have meaning to the girls’ wellbeing, and, in general, the wintertime was associated with feelings of discomfort as girls described winter as the time of physical isolation and a passive mood. It was not specified in this study what months of the wintertime wellbeing was perceived the lowest, but in the study of Konu et al. (2015), health status was perceived lowest from the middle of March to the end of May. Kristjánsdóttir et al. (2013) found that self-reported health in adolescent girls varied according to the season and its relation to medication and hormonal contraception. The girls’ descriptions of the effects of winter are also supported by the finding that the prevalence of Seasonal Affective Disorder (SAD) was also higher in female students, which was consistent with findings in previous research (Low & Feissner, 1998). This was also the case in the study of Tonettia et al. (2007), so it seems that seasonal sensitivity is higher in female subjects. In this study, the girls described temperatures between −20 to −30 °C as being too cold and making it inconvenient to go out. Thus, it is good to remember that cold results in concrete harm and that cold spells are associated with increased mortality rates in populations around the world (Ryti, Guo, & Jaakkola, 2015).

It is comprehensible that the girls described their wellbeing as being decreased during wintertime; coldness and darkness have restrictive impacts on girls and a limiting meaning to wanting to go outdoors. The reason for this was that walking in the darkness was experienced as being uncomfortable and gloomy, and the challenge with clothing also reduced the incentive to go outdoors. Perhaps as a result of the issues mentioned above, the girls in this study described being tired and reluctant during winter. Interestingly, Kronholm et al. (2015) report that insomnia symptoms and tiredness are more prevalent among girls in Finland. Borisenkov et al. (2015) demonstrate that significant phase delays of the sleep–wake rhythm and severe social jetlag were observed in females with winter depression, but not in males. They also state that both latitude of residence and location within the time zone are significant predictors of winter depression in young inhabitants of the North. Sourander et al. (1999) reported that seasonal changes in mood and behaviour were commonly reported among seventh and ninth graders, and during February and March, girls living in 67° N latitude reported more seasonal distress than girls living at 60° N latitude. But not only is the influence of latitude significant, other factors like climate, genetic vulnerability, and sociocultural context can be expected to play more important roles (Mersch, Middendorp, Bouhuys, Beersma, & van den Hoofdakker, 1999). Northern Finland is located between roughly 65° and 70° N latitude. Since the girls described wintertime as being challenging for their wellbeing, this could be eased with bright light therapy (Knapen, Werkena, Gordijna, & Meestersb, 2014).

This study attempts to contribute information to what the WHO (2014), in its European Child and Adolescent Health Strategy 2015–2020, wishes: to encourage the strengthening of people-centred health systems and public health capacity, in order to improve child and adolescent health development. As Kristjánsdóttir et al. (2013) reported, the health of adolescent girls needs to be understood against the background of their experiences of living conditions.

### Limitations

This research had some limitations. The first limitation relates to the sampling: although the 19 girls who participated in this study were from a wide range of different areas within Northern Finland, the girls who were willing to participate in this study may have been those who already perceived their health and wellbeing as being good. The study was done in a particular area which affects the transferability of the results. To ensure the intelligibility of the interview themes, a preliminary study was conducted by interviewing one adolescent girl and discussing with her the comprehensibility and the number of questions, as well as the duration of the interview. Although after the interviews, the researcher had a sense of data saturation, yet more interviews could have been done to ensure this.

The second limitation relates to the analytical process as the interviews were conducted, audio-recorded, and then transcribed verbatim by the researcher. Since the researcher's skills and experiences, as well as the scope and sensitivity of the problem (Elo et al., 2014; Polit & Beck, 2012), affect the research, this could pose a challenge. To document the trustworthiness of the analytical process, some of the girls’ original sentences are presented in the text after having been translated from Finnish into English.

The third limitation is that the analysis was not repeated independently by multiple researchers to determine the extent of agreement between separate analyses. However, the credibility of the findings was discussed within the research group (Elo et al., 2014). Two rounds of inductive content analysis were performed, and both of these yielded similar generic and main categories.

The fourth limitation is that the member checking technique—in which the results will be returned and discussed with the subjects—was not applied. But there is controversial information in this relevant practice (Polit & Beck, 2012).

The fifth limitation is that the average time of the interviews was around 21 min. Although the interviews were conducted until data saturation was obtained, the question arises whether the interviews had reached the depth necessary to illuminate the meaning of three different phenomena.

## Conclusions

The elements from the categories are interrelated and seem to have a meaning to girls’ wellbeing; the environment and its events (seasonal changes, nature, and animals) and occurrences are part of everyday life for girls. When perceiving challenging times or experiencing difficult conditions, which are the elements identified in this study that enhance wellbeing (e.g., walks in nature and interacting with animals), girls could be encouraged and advised to use these means. By utilising these elements, it seems that girls were seeking experiences and sensations that made them feel whole, accepted, and peaceful. Some of the challenges can be influenced by their own behaviour (e.g., the right to roam in nature, summer, and interactions with animals), which all seemed to contribute to adolescent girls’ wellbeing. Girls sought out liberating and rewarding feelings and stimulations to enhance their wellbeing. Walking in nature and interacting with animals seemed to offer premises for tranquillity and the possibility of sensory perceptions and comforting partnerships. Perhaps the empowering interactivity with animals gave positive experiences, functionality, and interaction; it seems that such interactions help the girls find themselves and thus improve self-esteem. This was also enhanced by the feeling of being accepted, as the girls are not being criticised or discriminated against. Based on the girls’ descriptions, animals as companions seem to reduce feelings of seclusion and isolation. Restorative nature compels the girls to live in the moment and thereby gives them perspective and makes them forget about unfortunate things for a while, which give them a sense of freedom. When girls were experiencing hardship or going through tough times, feelings of comfort and reward were gained from nature and animals. Involvement with nature seems to generate feelings of hope and vitality and create the sense of relatedness and belonging, thereby enhancing emotional wellbeing. Impairing effects are connected to winter and its related issues, such as coldness, darkness, the complexity of dressing correctly, fatigue, and reluctance. During wintertime, there seems to be a challenge for the girls to find balance for both their physical and mental wellbeing. Based on this study, girls living in Northern Finland should be supported in interacting with nature and animals. Although nature's restorative meaning on wellbeing seems to be relevant, girls’ wellbeing is also naturally influenced by other various factors such as friends, family, and feeling safe and optimistic. But in this study, the meaning of seasonal changes, nature, and animals to the adolescent girls’ wellbeing was described. The challenge for the future is perhaps the purposeful utilisation of nature and animals’ positive effects on wellbeing and the ethical questions related to them.

### Implications for practice and further research

From this perspective, girls living in Northern Finland are privileged, despite other debilitating factors of wellbeing. While living in the north exposes the girls to certain aspects that reduce wellbeing (darkness and cold), there are also elements that promote wellbeing, such as the benefits of nature and animals. Girls living in Northern Finland are fortunate because they have relatively easy access to nature's sphere of influence. This should be utilised and taken as a part of health and wellbeing promotion activities by offering girls to move safely in nature.

Based on the results of this study, having a pet may enhance girls’ wellbeing; hence, the acquisition of animals should be considered and supported. Since the girls described wintertime as being challenging for their wellbeing, we should consider how to respond to this. One way to meet this challenge would be that, during winter, the length of school days could be more flexible. Also, it has previously been known that bright light therapy and exercising may help to reduce negative emotional states. It would be good to have more information on the individual, social, and environmental factors that concern girls’ wellbeing in Northern Finland, including the participation and involvement of the adolescents themselves. Health promotion work that focusses solely on risk factors may lead to missing other important aspects that influence the adolescent girls’ health (Larsson, Sundler, & Ekebergh, 2012). Therefore, it would be relevant for further research to deepen the meaning of the findings from this study. To get more profound descriptions, it might be informative to use different research methods such as photo-elicitation and focus group interviewing. This could both enhance participation and help girls who cannot find words to describe the matters. Since wellbeing is not stable but varies according to different life situations and circumstances, effective promotion and prevention practices rely upon accurate and up-to-date information of the wellbeing of the girls.
